# Metabolic effects of influenza virus infection in cultured animal cells: Intra- and extracellular metabolite profiling

**DOI:** 10.1186/1752-0509-4-61

**Published:** 2010-05-13

**Authors:** Joachim B Ritter, Aljoscha S Wahl, Susann Freund, Yvonne Genzel, Udo Reichl

**Affiliations:** 1 Max Planck Institute for Dynamics of Complex Technical Systems, Bioprocess Engineering, Sandtorstrasse 1, 39106 Magdeburg, Germany; 2 Delft University of Technology, Bioprocess Technology, Julianalaan 67, 2628 BC Delft, the Netherlands; 3 Chair of Bioprocess Engineering, Otto-von-Guericke-University Magdeburg, Magdeburg, Germany; 4 Novartis Pharma AG, 4057 Basel, Switzerland

## Abstract

**Background:**

Many details in cell culture-derived influenza vaccine production are still poorly understood and approaches for process optimization mainly remain empirical. More insights on mammalian cell metabolism after a viral infection could give hints on limitations and cell-specific virus production capacities. A detailed metabolic characterization of an influenza infected adherent cell line (MDCK) was carried out based on extracellular and intracellular measurements of metabolite concentrations.

**Results:**

For most metabolites the comparison of infected (human influenza A/PR/8/34) and mock-infected cells showed a very similar behavior during the first 10-12 h post infection (pi). Significant changes were observed after about 12 h pi: (1) uptake of extracellular glucose and lactate release into the cell culture supernatant were clearly increased in infected cells compared to mock-infected cells. At the same time (12 h pi) intracellular metabolite concentrations of the upper part of glycolysis were significantly increased. On the contrary, nucleoside triphosphate concentrations of infected cells dropped clearly after 12 h pi. This behaviour was observed for two different human influenza A/PR/8/34 strains at slightly different time points.

**Conclusions:**

Comparing these results with literature values for the time course of infection with same influenza strains, underline the hypothesis that influenza infection only represents a minor additional burden for host cell metabolism. The metabolic changes observed after12 h pi are most probably caused by the onset of apoptosis in infected cells. The comparison of experimental data from two variants of the A/PR/8/34 virus strain (RKI versus NIBSC) with different productivities and infection dynamics showed comparable metabolic patterns but a clearly different timely behavior. Thus, infection dynamics are obviously reflected in host cell metabolism.

## Background

Yearly influenza epidemics with numerous death cases and severe economic impact demonstrate the urgent need for vaccinations against the flu. One characteristic problem for human influenza vaccination is the need for new vaccines every season because of the antigenic shift of the virus [1]. Various continuous cell lines capable of a virus replication to high titers are reported in literature [2,3]. Typically, the process consists of two stages: First, host cells are grown in bioreactors to cell numbers of 1-10 million cells per mL. Then, cells are infected with active virus, which replicates in the cells and finally new virus particles are released by a budding mechanism. Final product titers are clearly dependent on virus strains with some strains replicating only poorly in cell culture. Furthermore, the total product yield in upstream processing of vaccine production processes is low compared to other production processes using mammalian cells like antibody or recombinant protein expressions. Various hypotheses on the reasons for the low cell-specific productivity in general but also on strain dependence exist. Relevant mechanisms include antiviral response of host cells based on an interferon release, intracellular bottlenecks for virus replication and death rate of cells due to apoptosis [4-10]. Directly related to apoptosis is the metabolism of cells [11,12]. Especially the first enzyme of glycolysis, hexokinase, has been shown to be strongly involved in anti-apoptotic mechanisms interacting with voltage-dependent anion channels in the mitochondrial membrane, which is a major player in apoptosis [13].

Furthermore, apparently independent from apoptosis, central metabolism can be strongly affected by virus infections. A summary of early studies on metabolism of virus-infected animal cells is given by Koppelman & Evans [14]. Especially, transformations of cells after infection with tumor viruses are described to initiate an increased glucose metabolism and morphological changes [15-18]. In addition, a variety of other viruses have been shown to strongly influence host cell metabolism, e. g., rubella virus [19-21], cytomegalovirus [22-24], mayaro virus [25], dengue virus [26], mumps virus [27], newcastle-disease virus [27], polio virus [28-30] or reovirus [31].

Other studies report about the metabolic effects after influenza infections [32,33]. For an influenza virus infection of chick embryo fibroblasts, rates in glycolysis and pentose-phosphate pathway were elevated during early stages of infection [33]. The replication of virus requires energy for synthesis of macromolecules like proteins or, depending on the virus, DNA or RNA. Additionally, in the case of an enveloped virus, the budding of viruses forces cells to a *de novo *synthesis of membrane lipids. Calculations on energy needs for influenza virus production using MDCK cells based on virus composition estimated the demand for the production of 4000 virions per cell below 10% of the normal ATP turnover [4]. According to a different study based on a mathematical model, no limitations due to intracellular resources should occur if one cell produces about 8000 virus particles [7]. Calculation by metabolic flux analysis based on a model for MDCK cell growth [34,35] yielded an energy demand for virus production of only 2% of the normal ATP turnover to produce 6000 virions/h. The number has been estimated from HA titers from various experiments of our group (data not shown) with influenza virus (NIBSC) assuming that for the respective HA value the ratio of red blood cells to virus particles is 1:1.

Besides the obvious effects on metabolism caused by additional energetic needs for macromolecular synthesis, viral infections typically result in a variety of defence mechanisms within the host cell. On the other hand, reacting on the cell's strategies, viruses make use of various mechanisms to ensure reproduction. Strongly involved in virus replication are various complex mechanisms resulting in virus-induced apoptosis. Besides the above mentioned connection to the enzyme hexokinase, mitochondrial membrane breakdown at the final stage of apoptosis certainly results in severe consequences for metabolic homeostasis.

In this study, we focus on the analysis of the time course of a small number of important metabolites from central metabolism, because they can be highly informative and allow a clear distinction between metabolically different states. We present the investigation of influences of influenza virus infection on host cell metabolism by a quantitative metabolic profiling approach. A detailed time course of metabolic effects of infection of MDCK cells with two H1N1 influenza variants is described. Cells were infected at high multiplicity of infection (MOI ≥ 3) in order to achieve a synchronous infection. For comparison, a parallel mock-infection (without virus) was performed. During the following period of up to 34 h, adherent cells and cells in the supernatant were monitored. Extracellular and intracellular metabolite concentrations were measured at high sampling rates. A segregated model was developed to estimate the metabolic rates in the course of infection. Intracellular intermediates of glycolysis, tricarboxylic acid (TCA) cycle and nucleotides were assayed as indicators for metabolic changes of central metabolism. A two-phase spline approximation was used to determine the time of metabolic shift. Based on these measurements and calculations, clear conclusions could be drawn on direct and indirect effects of influenza virus infection.

## Results

### Infection experiments with the virus strain A/PR/8/34 (H1N1, RKI)

Three independent experiments were performed as described in the Methods section below. The sampling period after infection for the first, second and third experiment (Exp 1, Exp 2, Exp 3) were 22, 34 and 18 h pi, respectively. Mock-infection was omitted for Exp 3 to reduce experimental efforts. Due to technical or logistical difficulties, not all variables were measured for each experiment. For all experiments adherent and suspension cell numbers as well as extracellular metabolites glucose and lactate were determined. For the first and the second experiment (Exp 1; Exp 2) extracellular concentrations of glutamine, glutamate and ammonia and intracellular metabolite concentrations were measured additionally. Further extracellular amino acids were determined only for Exp 1. Virus titers were only measured for Exp 2 and Exp 3. For Exp 3 a high sampling frequency between 4 and 18 h pi was chosen. This resulted together with Exp 2 in a densely sampled time course during virus production.

As described in the Methods section, cell growth medium was exchanged by virus maintenance medium (VVM) at time of infection. Metabolic effects were to be expected due to this sudden change in substrate concentrations but also due to the switch to serum free conditions. To be able to distinguish between influences on metabolism caused by medium exchange and virus infection, a mock-infection was performed in parallel to infection (on the same 6 well plate). Since the differences between infected and mock-infected were of main interest, the metabolite concentration changes seen for the mock-infected cells will not be discussed in detail. Cells were infected with a MOI of 20 and a one-step, synchronous infection of cells was assumed.

### Cell numbers and virus titers

An inherent problem of infection experiments in static cultivation vessels, like six-well plates, is the determination of cell numbers. The main reason is the difficulty to detach all adherent cells before counting. The addition of trypsin to the VMM results typically in a deteriorated detachment behavior of cells compared to cell growth conditions. To compensate for this problem, a cell scraper was used for Exp 2 and Exp 3. Nevertheless, adherent cell numbers for infected cells showed a high variability. For this reason, cell numbers were taken from the model calculation. All cell specific values (rates and intracellular concentrations) are based on these cell number estimations.

**Figure 1:**
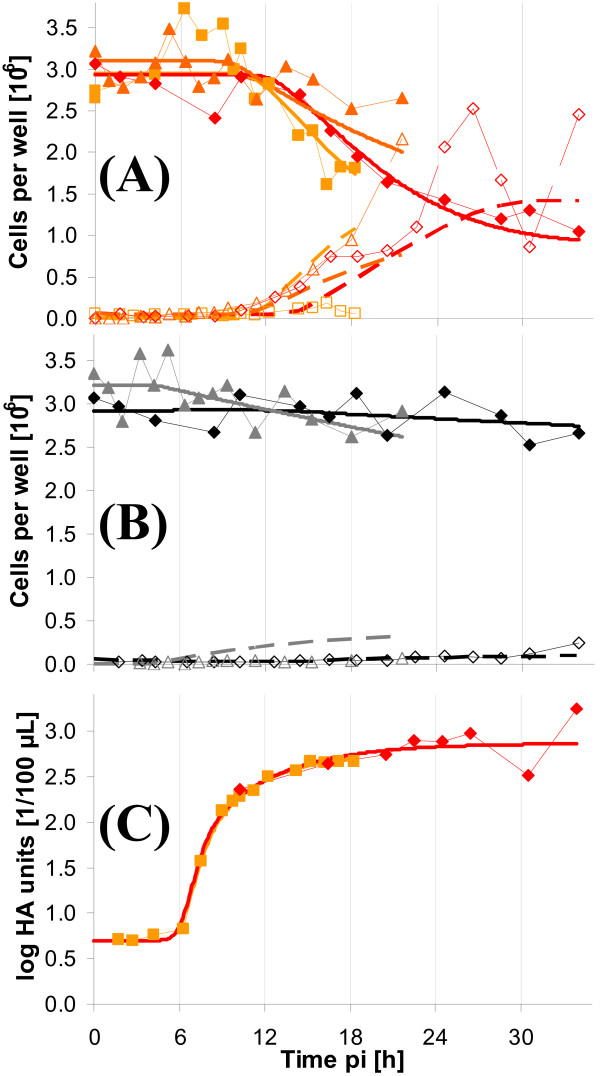
**Cell density and virus titers of MDCK cells after influenza resp. mock infection**. Three different experiments have been performed in six-well plates. The cells were infected with RKI (A) resp. mock-infected (B). Virus titers of infected cells of two experiments are shown in (C). Symbols represent average cell number from determination of three wells (Exp 1 △; Exp 2: ◇; Exp 3: □). Full symbols represent adherent cells, empty symbols represent detached (suspension) cells. Solid, thick lines indicate estimated cell numbers for adherent cells based on the mathematical model. Dashed thick lines represent the respective estimation for suspension cells. For (C): Symbols represent the average log HA value of three different wells. The thick line indicates the estimated HA-value according to the model. Data from HA measurements from the experiments RKI 2 and RKI 3. The fit for HA was only performed for RKI 2 (HA measurements from RKI 2 and 3 were combined).

Figure 1 shows the cell numbers for both, adherent and suspension cells as well as virus titers for experiments Exp 1-3 for infected (A) and mock-infected (B) cells. Thick lines indicate the estimated cell numbers and were calculated based on the modeling approach described in the materials section. According to our experience these interpolations reflect quite well the time course of cell numbers. Intracellular metabolite concentrations shown in later sections were calculated based on the estimated adherent cell numbers. Typically, cell numbers are constant for 6-8 h pi under the chosen experimental conditions. Accordingly, approximations of cell numbers were also constant. Starting cell numbers at time of infection, however, varied for the different experiments. Although the number of cells at the time point of infection was determined, it was additionally estimated by the modeling approach (infection/mock-infection were treated as independent experiments). Overall, estimated cell numbers either decreased slowly for infected cells after about 10 h pi (Exp 1), or comparatively fast, as in Exp 3. After 30 h pi, adherent cell numbers had dropped to about 1 × 10^6 ^cells per well (Exp 2) from an initial amount of 3 × 10^6^. Mock-infected cell numbers were comparatively constant until the end of experiment.

Total loss in cells for mock-infection did not exceed more than approximately 10% of the initial cell number. In summary, the time course of cell numbers was clearly different for infected and mock-infected cells.

Slightly preceding the beginning of cell detachment in infected cultures, HA-values started to increase as shown in Figure 1(C). The final HA titer was about 1800 HAU/100 *μ*l for the longest experiment (Exp 2).

### Extracellular metabolite concentrations

**Figure 2:**
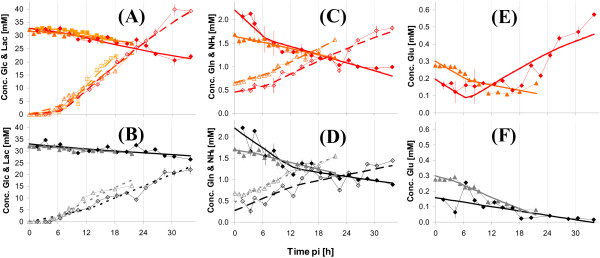
**Concentrations of extracellular metabolites for MDCK cells after infection with influenza virus**. Measurements from infections with the RKI variant are shown in the first row, mock-infection in the second row. Symbols represent average concentration from determination of three wells (Exp 1 △; Exp 2: ◇; Exp 3: □). (A) & (B): glucose (full symbol, solid lines) and lactate (empty symbol, dashed lines), three experiments; (C) & (D): glutamine (full symbol, solid lines) and ammonia (empty symbol, dashed lines), two experiments; (E) & (F): glutamate, two experiments. Thick lines indicate the estimation of the concentration based on the mathematical model.

Due to medium exchange at time of infection, glucose, glutamine and glutamate concentrations were high at the beginning of the infection period and decreased according to the metabolic activity of cells (Figure 2). Mock-infections revealed similar time courses within the first 10 h pi. Lactate concentrations were below detection limit within the first 5 h pi for mock-infected and infected cells. At about 6 h pi, a steep increase in lactate concentrations was measured, which, however, seemed to be identical for mock-infected and infected cells until about 10 h pi. After approximately 10 h pi, glucose uptake as well as lactate release of infected cells strongly increased indicating a change in glycolytic activity.

Concentration profiles of glutamine were basically identical for mock-infected and infected cells and no significant differences could be detected. Also, for ammonia, no obvious differences could be seen.

The concentration of glutamate (Figure 2C), revealed the well known pattern [36] of an initial consumption of glutamate within the first 10 h pi and a subsequent strong release of glutamate of infected cells. While the behavior for mock-infected cells was identical within the first 10 h pi, no increase in concentration of glutamate was observed in the following period.

Using the segregated model (see Methods section), specific rates for uptake and excretion of extracellular metabolites for the two metabolically different cell types of the model were estimated. Table 1 summarizes these results. For infected cultures, the specific rates of metabolically modified cells (definition given in the methods section) are given, and for mock-infected cultures the rates of the non-infected cells.

**Table 1:** Specific uptake and release rates of extracellular metabolites of infected (variant from RKI) and mock-infected cells.

	Cell specific rate [fmol/cell/h]					
	**Glucose**		**Lactate**		**Glutamate**	

	***infected***	***mock-infected***	***infected***	***mock-infected***	***infected***	***mock-infected***

Exp 1	-93	-52	510	339	-1	-4
Exp 2	-158	-45	729	295	11	-2
Exp 3	-193	n.d.	986	n.d.	13	n.d.

Again, clear differences between infected and non-infected cells were detected (Table 1) at about 10 h pi. In particular, specific glucose uptake and lactate release rates of infected cells were strongly increased compared to mock-infected cells. Interestingly, significantly more lactate was released from infected (and metabolically modified) cells than expected from degradation of extracellular glucose (see discussion). Specific rates for glutamate were mainly positive for infected cells (release), while mock-infected cells showed a negative rate (uptake). An exception was Exp 1 during which glutamate was also taken up from infected cells.

**Figure 3:**
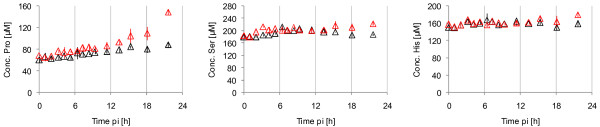
**Concentrations of selected extracellular amino acids for MDCK cells after infection with influenza virus**. Symbols represent average concentrations from three wells of the experiment with the RKI variant (red) and mock-infection (black) for Exp 1. Error bars represent the standard error of the mean.

Additionally to glutamine and glutamate, 15 amino acids (Arg, Asn, Ala, Thr, Gly, Val, Ser, Pro, Ile, Leu, Met, His, Phe, Asp, Cys) were measured by anion-exchange HPLC for Exp 1. Infection resulted in a release of the amino acids Pro, Ser and His compared to mock-infection (see Figure 3). For several other amino acids, a clear uptake (e.g., Gly, Asp, Met) or release (e.g., Asn, Ala, Thr) was measured, but this occurred for both infected and mock-infected cells. Therefore, all other amino acid concentrations did not show any clear tendency as an effect caused by infection.

### Intracellular metabolite concentrations

**Figure 4:**
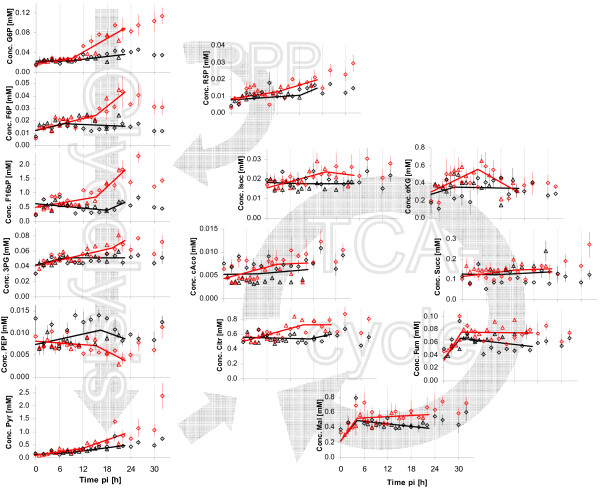
**Intracellular metabolite concentrations after infection**. Symbols represent average concentrations of three wells of one six-well plate, the error bars the respective standard error of the mean. Two different experiments were performed under identical conditions (Exp 1 △; Exp 2: ◇). Solid lines indicate linear spline interpolations for two phases performed with combined data from both experiments for data points up to 22 h pi.

Intracellular metabolite concentrations were determined by anion-exchange chromatography after sample preparation (see Methods, Determination of intracellular metabolite concentrations). Focus was on central metabolism due to general relevance of the corresponding pathways for viral precursor synthesis. All metabolites quantified are summarized in Table 2. The time course of metabolites from glycolysis, pentose-phosphate-pathway (PPP) and TCA-cycle from Exp 1 and Exp 2 is shown in Figure 4. Solid lines indicate the interpolation of data points based on the two-phase linear spline approach (see Methods). Experimental data of both experiments were interpolated in a combined way. To avoid weighting of data points, and also because the last two data points of Exp 2 indicated new tendencies, only data points of comparable time ranges were used, i.e., the points of Exp 2 later than 22 h pi were omitted for the spline fitting.

**Table 2:** Overview of quantification methods used for intracellular metabolite determination.

Metabolite	Detector	Calibration type	Concentration in standard stock [*μ*M]
**3PG**	ECD	QOff	25
**ADP**	UV	LOff	200
**AMP**	UV	XLOff	100
**ATP**	UV	LOff	400
**CDP**	UV	QOff	25
***cis*-Aconitate**	MS	XQuad	25
**Citrate**	ECD	QOff	250
**CMP**	UV	LOff	25
**CTP**	UV	LOff	40
**Fructose-1,6-bP**	ECD	XQOff	228
**Fructose-6-P**	MS	QOff	35
**Fumarate**	UV	XLOff	50
**GDP**	UV	LOff	40
**Glucose-6-P**	MS	XQOff	35
**GMP**	MS	XQuad	25
**GTP**	UV	LOff	79
**Isocitrate**	MS	XQuad	25
**Malate**	MS	Quad	200
**PEP**	MS	XQuad	25
**Pyruvate**	MS	Quad	200
**Ribose-5-P**	MS	XQuad	25
**Succinate**	MS	XQOff	200
**UDP**	UV	LOff	25
**UDP-Glc**	MS	XQOff	70
**UDP-NAc-Gal**	UV	XLOff	60
**UDP-NAc-Glc**	UV	XLOff	200
**UMP**	MS	XLOff	75
**UTP**	UV	Lin	200
***α*-Ketoglutarate**	MS	XQOff	100

Lin: Polynomial 1. Ord. forced through origin

LOff: Polynomial 1. Ord. NOT forced origin

XLOff: Polynomial 1. Ord. NOT forced origin, weighted *(1/concentration)*

Quad: Polynomial 2. Ord. forced through origin

QOff: Polynomial 2. Ord. NOT forced through origin

XQuad: Polynomial 2. Ord. forced through origin, weighted (*1/concentration*)

XQOff: Polynomial 2. Ord. NOT forced through origin, weighted *(1/concentration)*

### Intermediates from glycolysis and pentose-phosphate pathway

Several metabolites from glycolysis (glucose-6-phosphate (G6P), fructose-6-phosphate (F6P), fructose-1,6-bisphosphate (F16bP), 3-phosphoglycerate (3PG)) and pentose-phosphate pathway (ribose-5-phosphate, (R5P)) showed a very similar pattern. Within the first 10 h pi for both infected cells and mock-infected cells concentrations were constant. For infected cells a linear increase in concentration was measured after 10 h pi until about 24 h pi. After this time point, concentrations of G6P and R5P increased until the end of the investigated period for infected cells. In contrast, the concentrations of the metabolites F6P, F16bP and 3PG decreased after 24 h pi for infected cells, but remained higher than for mock-infected cells. An exceptional concentration behavior was observed for phosphoenolpyruvate (PEP). Concentrations were almost identical for infected and mock-infected cells within the early infection phase and dropped in infected cells below the values in mock-infected cells after approximately 10 h pi. The final metabolite of glycolysis, pyruvate, also showed no concentration differences within the first 10 h pi for infected and mock-infected cells. After 10 h pi, pyruvate concentrations of infected cells clearly increased, similar to the behavior of the first glycolytic metabolites.

### TCA-cycle intermediates

In addition to metabolites from glycolysis, several metabolites from TCA-cycle were quantified. Citrate, isocitrate and *cis*-aconitate showed qualitatively very similar concentration time courses. No differences in concentrations could be observed within the first 10 h pi. From about 10 h pi up to 14 h pi, concentrations in infected cells increased, whereas concentrations in mock-infected cells remained fairly constant. From 14 h pi to the end of experiments the concentrations for both infected and mock-infected cells remained constant with infected cells having slightly higher concentrations.

Concentrations of *α*-ketoglutaric acid were comparable within the first 6 h pi in infected and mock-infected cells. In the following 12 h concentrations in infected cells appeared to be higher than in infected cells. Approaching the end of the investigated period, the concentration of *α*-ketoglutaric acid in infected cells decreased to the level of mock-infected cells. Concentrations of succinate did not reveal any major differences between infected and mock-infected cells up to 24 h pi. In samples after 24 h pi, slightly higher succinate concentrations were measured in infected cells. The effects of virus infection were very similar for fumarate and malate. During the first 6 h pi in both infected and mock-infected cells concentrations increased in an identical manner. In the following period of the experiment, concentrations were higher in infected cells than in mock-infected cells

### Nucleotides

**Figure 5:**
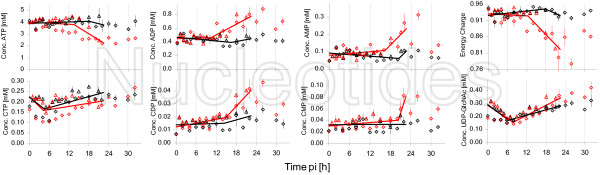
**Intracellular concentrations of nucleotides and energy charge after infection**. Symbols represent the average concentrations of three wells after infection with RKI (red) resp. mock-infection (black). Two different experiments were performed under identical conditions(Exp 1 △; Exp 2: ◇). Solid lines indicate linear spline interpolations for two phases performed with combined data from both experiments for data points up to 22 h pi.

Metabolites of great physiological importance are nucleotides. Figure 5 shows selected intracellular nucleotide concentrations and, additionally, the energy charge (EC = ([ATP] + 1/2 [ADP])/([ATP] + [ADP] + [AMP])).

The majority of nucleotides showed an indistinguishable concentration behavior for infected and mock-infected cells for at least the first 8-10 h pi. Thus, this period will not be described in detail in the following sections.

A clear decrease in concentration of ATP in infected cells was observed in both experiments in the late phase of the experiments, even though the start of concentration decrease did not appear simultaneously (Exp 1: 18 h pi; Exp 2: 12 h pi).

As expected, ADP and AMP showed for infected cells an inverse concentration behavior to ATP. Concentrations increased clearly after about 12 h pi with a maximum value at about 24 h pi. However, concentrations were decreased again after 30 h pi and were in comparable concentration ranges as for mock-infected cells.

As an example for pyrimidine nucleotides, cytidine tri-, di- and monophosphate concentrations are shown in Figure 5. The impact of infection on CTP concentrations did not appear to be of the same magnitude as for ATP. Although concentrations were lower in infected cells after about 12 h pi, the differences to mock-infected cells were of minor extent. Concentration differences between infected and mock-infected cells disappeared towards the end of the experimental period and concentrations in infected cells even exceeded those in mock-infected cells. Concentrations of GDP and GMP showed a similar behavior as those of ADP and AMP, i.e. concentrations increased (data not shown). Remarkably, for both adenosine and cytidine nucleotides, intracellular concentrations of diphosphates started increasing first and, with a delay of about 6 h, monophosphates started increasing. Contrarily to the time course of nucleoside triphosphates, the measured diphosphates did not indicate a time gap larger than about 4 h between the two different experiments, i.e. the increase started for both experiments approximately at the same time.

As another variable, the EC is shown in Figure 5. The EC dropped for both experiments clearly after an initial period of about 12 h pi. The time gap for the two experiments for this variable was in between that for nucleoside triphosphates and mono- and diphosphates, i.e. about 6 h. The decrease of the EC resulted in values below 0.85 for both experiments for infected cells, whereas the EC for mock-infected cells remained at constant values of over 0.92.

One relevant metabolite with respect to protein glycosylation is UDP-GlcNAc. Although starting concentrations for the two different experiments were not identical, the qualitative course of concentrations was comparable. Initially, concentrations decreased for both infected and mock-infected cells. After about 10 h pi, concentrations started rising, whereas the slope of the increase was steeper for infected cells.

### Timing of metabolic switches

**Figure 6:**
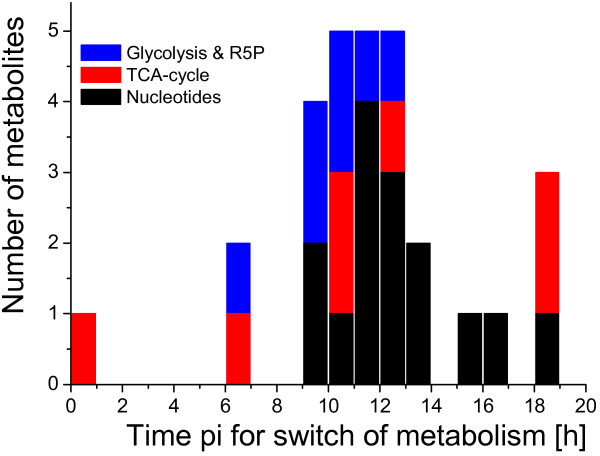
**Histogram of time points for the metabolic switch for quantified intracellular metabolites**. In total, 29 metabolites are summarized and grouped according to metabolite class. Glycolytic metabolites & R5P are blue; intermediates from TCA-cycle are red; nucleotides are black. Binning was set to 1 h.

Even though it is possible to assess time points of metabolic changes between infected and mock-infected cells based on the shown intracellular metabolite profiles, further calculations were performed to yield a more precise estimate of the overall time point for the switch of metabolism. Therefore, intracellular concentrations of mock-infected cells were subtracted from concentrations of infected cells. Thus, concentration changes occurring simultaneously in both infected and mock-infected cells were compensated and the switch from equal behavior of mock-infected cells and infected cells to different behavior could be recognized by the spline approximations (see Methods section). Based on these calculations, an individual time point for each metabolite indicating the metabolic switch could be identified. In principle, these time points corresponded well with the visual assessments based on the respective concentration profiles. The distribution of switch-points is shown in Figure 6, a histogram of time points pi for the different metabolites.

The calculated average time of metabolic switch for all 29 metabolites included was 11.6 h pi. While metabolites of TCA-cycle showed very inhomogeneous switch-points from 1 h pi up to 19 h pi (average 11.1 h pi), metabolites from glycolysis had switch-points in a narrow window from 6-13 h pi (average 9.9 h pi). Nucleotides appeared to be metabolically affected later, having an average switch-point of 12.6 h pi. The very low and very high switch-times in Fig. 6 seem to result from extreme cases in which the detection of the switch-time reacts very sensitively to measurement noise and outliers. In these cases there are (i) only very small concentration differences between infected and mock-infected cells and (ii) extreme values at the begin (here for isocitrate) or the end (here for CTP, citrate, *cis*-aconitate) of the experiment. Nevertheless the estimation was stable and in the expected time window for the great majority of metabolites (25 out of 29).

Summarizing the results of intracellular concentration measurements it is obvious that influenza virus infection affects most metabolites of glycolysis and TCA-cycle of MDCK cells. However, the magnitude, the direction, and timepoints of the effects strongly differed.

### Infection experiments with the virus strain A/PR/8/34 (H1N1, NIBSC)

For the identification of a more general pattern concerning the effects of an influenza virus infection on host cell metabolism, further experiments with a different virus strain were carried out. The virus variant of A/PR/8/34 H1N1 from NIBSC was used because of the significant differences in replication dynamics compared to the variant from RKI [10]. Typically, infection of MDCK cells with the virus from NIBSC resulted in fast induction of apoptosis and an early cell death. Interestingly, virus titers were often lower for this variant compared to the variant from RKI. Here, it was investigated whether the fast advance of infection also leads to a faster response in host cell metabolism. Infections were performed with a MOI of 3. In contrast to the RKI experiments at this MOI 20% of the total cultivation volume consisted of seed virus due to the low TCID_50 _titer of the seed virus

**Figure 7:**
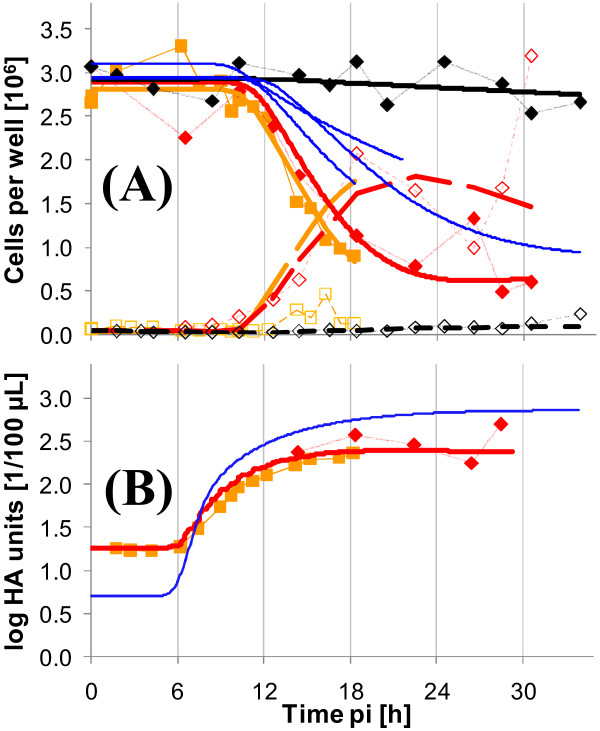
**Adherent and suspension cell density after infections with influenza variant from NIBSC**. Two different experiments where performed with NIBSC variant (red symbols and lines, Exp 2: ◇; Exp 3: □) and mock-infection (black symbols and lines) for Exp 2 in six-well plates (A). Virus titers of infected cells (B). For further explanation it is referred to Figure 1. Additionally, blue lines indicate the estimates of the respective variables for the RKI variant (see also Figure 1).

Infections with this influenza variant were performed in parallel with infections with the RKI variant of Exp 2 and Exp 3. Only one data set for mock-infection will be shown here as no mock-infection was done for Exp 3 and the shown data set is identical with the mock-infection of the data from mock-infection shown in the context of infections with the RKI virus. In Figure 7(A), adherent and suspension cell numbers are depicted for the two experiments for infected cells and for the mock-infected cells of Exp 2. Additionally, virus titers are shown in Figure 7(B).

**Figure 8:**
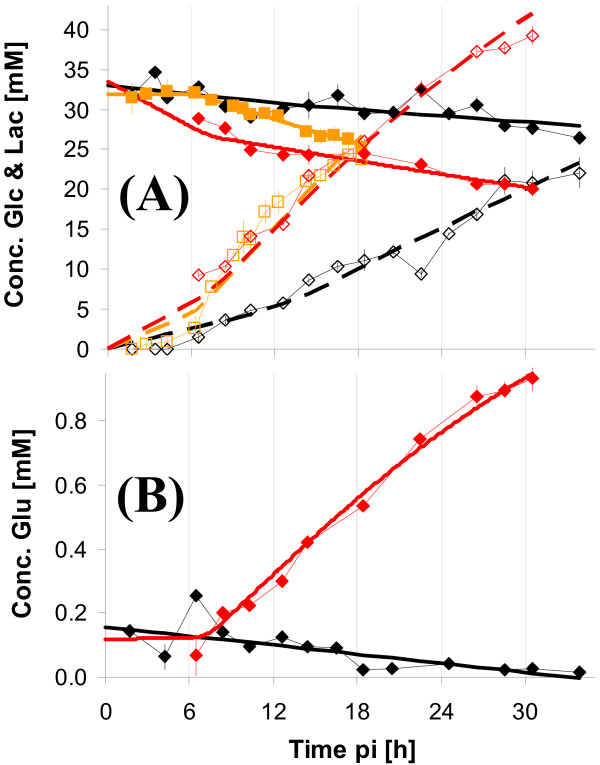
**Concentrations of extracellular metabolites after infection with influenza virus from NIBSC**. MDCK cells were infected with NIBSC virus (red symbols and lines) or mock-infection (black symbols and lines). Symbols represent average concentrations from determination of three wells. (A): Glucose (full symbol, solid lines) and lactate (empty symbol, dashed lines), two experiments (infection: Exp 2: ◇; Exp 3: □; mock-infection: Exp 2); (B): glutamate (Exp 2). Thick lines indicate the estimation of concentration based on the mathematical model.

Cell number of adherent cells decreased quickly after about 10 h pi approaching asymptotically a final value of surviving cells of about 5 × 10^5 ^cells/well until the end of the investigated time span. Fitted curves for both sets of experiments agreed well giving a similar estimate of the time course of cell numbers. Virus titers increased strongly after 6 h pi. Similar to the infection with the virus from RKI, the NIBSC virus induced clear changes in uptake and release rates of extracellular metabolites (Figure 8). Rates for lactate and glutamate were altered most significantly. But also glucose was consumed faster after infection with virus from NIBSC compared to mock-infection. Rates of glutamine and ammonia were not changed after infection compared to mock-infection and therefore the respective concentrations are not shown. At the first sampling point after 6 h pi pronounced differences in the lactate concentration were already measured. Similar results were obtained for glucose concentrations. Higher glutamate concentrations, however, were not measured earlier than 8-10 h pi.

A comparison of cell specific rates for uptake or release of extracellular metabolites for infected and mock-infected cells based on the above mentioned modeling approach clearly underlined the finding of an altered metabolism of infected cells (see Table 1). Glucose uptake and lactate and glutamate release were significantly increased in infected cells.

As already described for the RKI variant, the carbon-balance could also not be closed for the infections with the NIBSC variant. Reasons for this inconsistency will be discussed later.

**Figure 9:**
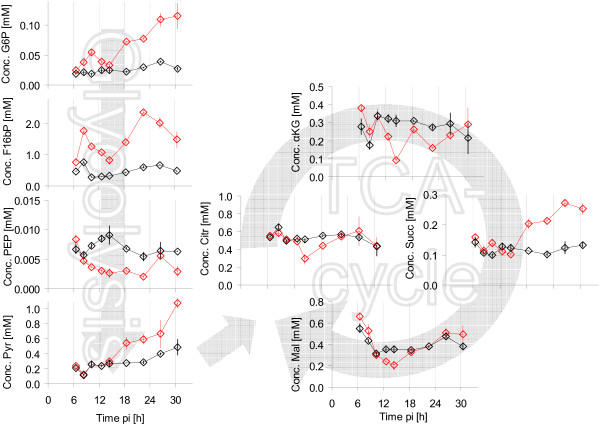
**Intracellular concentrations of metabolites**. Time course of intracellular metabolites from glycolysis, TCA-cycle after infection with influenza virus from NIBSC (red) and mock-infection (black) for Exp 2. Symbols represent average concentrations of three wells of one six-well plate and error bars the respective standard error of the mean.

The comparison between infected cells and mock-infected cells concerning intracellular concentrations of metabolites from glycolysis and TCA-cycle revealed a very similar pattern as seen for the infection with virus from RKI. Some representative intracellular concentrations of metabolites from glycolysis and TCA-cycle are shown in Figure 9.

The concentration behavior of glycolytic intermediates revealed for cells infected with the NIBSC variant a comparable pattern to infections with the RKI variant. The initial period of indistinguishable concentrations between infected and mock-infected cells, however, was shorter. Thus, first concentration differences between infected and mock-infected cells were measured already after 8 h pi (e.g., F16bP). A further difference to the effects caused by the virus from RKI was that a local minimum occurred after the first increase in concentrations of intermediates from upper glycolytic part.

The concentration of 3PG showed slightly higher values for infected cells after about 18 h pi (not shown). PEP concentrations were lower in infected cells compared to mock-infected cells, similar to the effect caused by the infection with the virus from RKI. Pyruvate concentrations of infected cells were higher compared to mock-infected cells.

With respect to intermediates of TCA-cycle, no uniform pattern could be recognized compared to the effects caused by the RKI variant. Four organic acids are shown in Figure 9. Metabolite concentrations not shown behaved similarly (isocitrate and *cis*-aconitate resembled citrate; fumarate resembled malate). Until 10 h pi, no obvious differences between infected and mock-infected cells were measured, independent from metabolite. Within the period from 10 h pi up to 18 h pi, concentrations were slightly lower in infected cells. Final concentrations of investigated organic acids from TCA-cycle were very similar for infected and mock-infected cells with exception of succinate. Significantly higher concentrations were measured for succinate in infected cells compared to in mock-infected cells.

**Figure 10:**
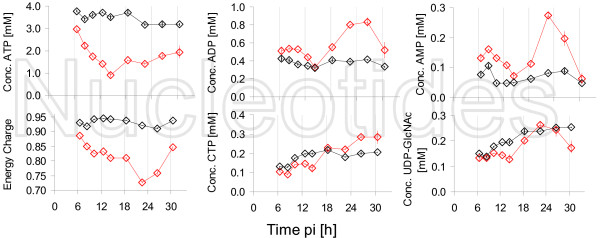
**Intracellular concentrations of selected nucleotides and energy charge after infection with influenza virus from NIBSC**. Infection was performed with NIBSC (red) and mock-infection (black). Symbols represent average of three wells of one six-well plate and error bars the respective standard error of the mean.

Additionally, nucleotides were quantified for this experiment. Selected nucleotides and the EC are depicted in Figure 10.

The concentration of ATP revealed a strong decrease starting with the first sampling time point. After 14 h pi, a minimum concentration was reached followed by a slight increase until the end of the experiment. The course of concentrations of ADP and AMP resembled each other to a high degree. While concentrations in mock-infected cells remained fairly constant, concentrations in infected cells initially increased up to about 10 h pi and a decrease followed with minimum value at about 16 h pi. Another concentration increase followed up to about 24 h pi. Until the end of the experiment, concentrations dropped to values close to values of mock-infected cells. As expected from the time courses of adenosine nucleotides, the EC was constant for mock-infected cells and was significantly lower for infected cells. The minimum value for the EC after about 22 h pi was below 0.75. For the metabolite UDP-GlcNAc, no clear differences were found between infected and mock-infected cells.

Summarizing the data obtained for infections with the virus from NIBSC clearly shows that main trends were similar to those for infections with the RKI virus. Time points of differences between infected and mock-infected cell metabolism, however, appeared shifted to earlier time points after infection for the NIBSC variant compared to the RKI virus.

## Discussion

### Cell numbers and virus titers

Although a standardized procedure for cell counting was used, comparably high variations were observed. To compensate for this variability, replicated measurements (3 to 12×) were performed. As mentioned above, main reason certainly is the changed trypsinization behavior of cells for cell number determination in VMM due to supplementation of medium with trypsin. Further reasons for the variability for the individual time points are most probably a slightly different handling by different persons sampling, but also non-identical cell behavior within and between six-well plates. Both cell growth and virus infection are stochastic events resulting in a distribution of physiological states for different wells, whereas multiple factors influence the width of this distribution.

A segregated modeling approach was applied that leads to a smoothing of time course of experimental data based on balance equations. Therefore, only certain changes are possible from one sampling time point to the next. All measurements influence the parameter estimation, which compensates for some of the variances. The estimation of cell numbers based on the model indicated good agreement for the different experiments (see Figure 1 and Figure 7) underlining the reproducibility of the experimental design.

Time courses of cell concentrations and virus titers showed good agreement with the work published by Schulze-Horsel et al. (2009). The same virus variants were investigated and a qualitatively similar behavior was observed, i.e. the virus from NIBSC led to faster cell death and produced higher virus titers [10]. While in our work, however, the first measurable virus release occurred at about the same time for both virus variants, Schulze-Horsel et al. found an earlier virus release for NIBSC. Main reason might be that, in contrast to our work, Schulze-Horsel et al. infected cells with a low MOI (0.025). Moreover, cultivation systems were different (six-well plates versus bioreactor).

### Extracellular metabolite data

Extracellular metabolite concentrations (see Figure 2 for RKI virus and Figure 8 for NIBSC virus) of infected cells revealed qualitative typical patterns for the influenza virus production process, independent from cultivation system for both virus variants [36-38]. Typically, glucose, glutamine and glutamate consumption as well as lactate and ammonia release are observed within the first hours after virus infection. In the late phase of virus replication, a significant increase in glutamate concentration was normally observed [36-38]. Depending on the experiments, extracellular concentrations varied in a normal range (RSD of 15%) caused by measurement and pipetting errors as well as by the biological variance (see error bars Figures. 2 & 8). One difficulty with the extracellular measurements for both infected and mock-infected cells was the impossibility to close the carbon balance. This means that higher amounts of lactate were released from cells than the maximum theoretical yield from glucose should have allowed. A common problem of cultivations in six-well plates is evaporation, also in humidified incubators. We measured evaporation to be approximately 2.75 *μ*L/h/well during typical cultivation experiments. This value, however, could not account completely for the gap in the carbon balance. Retrospectively, it was concluded that evaporation was probably higher for the shown experiments than for typical cultivations, because high sampling frequency demands the incubator had to be opened many times. As humidity drops every time the incubator is opened increased evaporation from plates is to be expected. Nevertheless, even assuming higher evaporation losses can not completely explain the observed lactate to glucose ratios.

A further error could arise from the used measurement device (Bioprofile 100 Plus, see Methods section). The calibration of the enzymatic sensors is performed in serum-containing GMEM medium. There could be a small difference in the enzymatic reaction conditions leading to an overestimation of lactate. But, the observed deviation is much higher than a possible calibration shift due to slightly different media.

Taking into account the uptake of amino acids in addition is also not sufficient to explain the remaining fraction of lactate released. Therefore the non-closable carbon balance remains an open question for these experiments unless assuming that the high values for lactate excretion are related to the degradation of endogenous substrates like glycogen or proteins. Since the non-closable C-balance occurred for infected and mock-infected cells, it is not related to the infection process but rather an effect of the cultivation conditions chosen. As the main focus of this study was on differences in metabolism between infected and mock-infected cells, no further effort was put into an elucidation of this phenomenon.

Infection was performed using a highly concentrated seed virus solution (modified protocols) to keep metabolic influences of spent medium of virus seed as low as possible. While we assume that for the RKI variant, the influences of the 2% of used medium could be neglected, the 20% of used medium of the virus stock solution for the NIBSC variant might have had certain metabolic influences. But due to the fact that general tendencies were the same for both virus variants, we are confident that main trends were effects of viral infection and not caused by the medium.

### Segregated mathematical model

A modeling approach was used to smooth experimental data and to estimate cell numbers, specific metabolic rates and time points of metabolic switches. A segregated model was used to differentiate the specific rates of different cell populations (uninfected, infected, infected and metabolically modified, detached cells). The metabolic activity in each cell population was assumed to be at quasi-steady state conditions. Thus, the time needed for a change in metabolic activity in the transient from one state to the other was assumed to be short compared to the duration of experiments. Because some populations cannot be distinguished directly from the measurements and still low sampling rate (for a deconvolution approach) the specific rates of cell populations have been lumped: uninfected and infected, as well as metabolically modified and detached cells. Thus, the resulting specific rates are averaged rates over lumped populations and the respective time. Despite these simplifications the identification of single population rates based on measurements of all cells metabolic activities (uninfected, infected and metabolically modified as well as detached) is rather ill posed and leads to highly correlated parameters. Therefore transition rates have been estimated based on all available data and then fixed in the single data-set fitting. Because of the non-closed carbon balance, the specific uptake and excretion rate were not consistent with a maximal Lac/Glc ratio. Nevertheless the model estimations show a clear increase in uptake and excretion rates of infected cells compared to mock-infected ones. Also, the model simulations were able to well reproduce the measurements within the measurement errors. An overview of the estimated parameters is given in Table 3.

**Table 3:** Estimated parameter values for the different experiments (RKI, NIBSC, mock).

	Virus infection					Mock	
			
	*RKI1*	*RKI2*	*RKI3*	*NIBSC1*	*NIBSC2*	*Exp. 1*	*Exp. 2*
*X*_*uinf *_(0)	3.10	2.91	2.95	2.87	2.81	3.21	2.92
*X*_*det *_(0)	0.17	0.25	0.0	0.36	0.0	0.0	0.05
*X*_*ui *_(10^6^/mL)	0.70	0.93	1.10	0.70	0.67		

*r*_*lys *_(1/h)	0.03	0.03	0.03	0.03	0.03	0.03	0.03
*r*_*inf *_(1/h)	5	5	5	5	5		
*r*_*mm *_/*r*_*adp *_(1/h)	0.70	0.70	0.70	0.80	0.80	1.0	1.0
*r*_*det *_(10^-3^/h)	44.1	76.3	100.5	109.6	135.2	11.8	3.0

*τ*_*inf *_/*τ*_*adp *_(h)	6.3	6.3	6.4	5.2	5.7	4.0	10.0
*τ*_*det *_(h)	3.0	5.7	4.2	5.1	4.4		

*r*_*Glc*,1_	-28.5	-70.9	-18.5	-403.5	0.0	-33.9	-61.1
*r*_*Glc*,2 _(fmol/cell/h)	-93.0	-157.5	-192.9	-66.0	-255.4	-51.8	-45.0

*r*_*Lac*,1_	124.5	1.5	69.2	177.6	252.1	43.9	140.8
*r*_*Lac*,2_	509.8	728.8	985.9	666.9	809.9	338.6	295.3

*r*_*Glu*,1_	-5.0	-6.7	-9.4	-10.0	-10.4	-2.6	-1.6
*r*_*Glu*,2_	-0.8	10.5	12.6	20.4	16.5	-4.3	-1.7

*r*_*Gln*,1_	-6.2	-31.1	-0.2	-40.1	-0.5	-5.5	-26.1
*r*_*Gln*,2_	-7.4	0.0	-3.6	0.0	-5.9	-9.3	-5.6

*r*_*NH*4,1_	7.8	5.5	2.8	3.2	2.2	20.8	16.0
*r*_*NH*4,2_	17.4	19.7	8.0	19.7	10.5	15.5	7.8

*r*_*VP*,2 _(VP/Cell/h)		27500	30800	9600	9300		

*Glc*(0) (mmol/L)	31.6	32.7	32.7	33.8	31.9	32.2	32.9
*Lac*(0)	0	0	0	0	0	0	0
*Glu*(0) (*μ*mol/L)	298.5	195.7	315.0	175.7	362.7	298.2	157.3
*Gln*(0)	1.6	2.2	0.8	2.1	0.8	1.7	2.2
*HA*(0) (logHA)		0.7	0.7	1.3	1.3		
*NH4*(0)	648.4	445.5	191.3	847.5	238.2	466.2	265.5

### Metabolic rates

Whereas within the early phase of infection no metabolic differences between infected and mock-infected cells were measured, glycolytic activity was clearly increased after 12 h pi for cells infected with the virus from RKI and after about 8 h pi for the NIBSC variant. This is especially reflected in cell specific rates (see Table 1 & Table 4). An elevated rate of glycolysis was observed before for various viruses [15-18,20-25,27,30], including influenza virus [14,33]. Klemperer (1961) could show for influenza infected chick embryo fibroblasts within the first 1.5 h pi after a synchronous infection increased glucose uptake and lactate release rates as well as an enhanced activity of the pentose-phosphate pathway [33]. This is in contrast to the work presented here, where extracellular effects were measured only after 8 h pi. With high probability, part of this difference in metabolic behavior is related to the specific cell line and the virus strain used in the work presented here compared to the work by Klemperer (1961) [33]. In addition, there were methodological differences concerning infection and cultivation procedures as well as assays used. Klemperer concluded from his results that the infection with virus causes direct metabolic adaptations for synthesis of new virus particles [33]. This is in contrast to theoretical considerations on energetic requirements by Genzel et al. (2007) and Sidorenko et al. (2004), which suggested that intracellular virus replication does not have a major impact on the cellular ATP balance [4,7]. Also calculations based on a metabolic flux model [34,35] suggest that cellular ATP needs for virus synthesis does not exceed 2% of the total ATP turnover during the stationary cell growth phase (not shown). In contrast to the work of Klemperer [33], Fisher and Ginsberg [32] found that glycolysis was reduced within a short period of time after virus addition. Experimental conditions, however, differed to a great extent regarding host cells and virus strain. Especially the fact that influenza virus only adsorbs to leucocytes but does not replicate makes a comparison of these two studies difficult. Nevertheless, both publications demonstrate that most probably different metabolic effects on the cells are to be expected after influenza infection and moreover, that host cells might react in different ways to influenza infections. Thus, it is not surprising that MDCK cells infected with different virus strains and under different conditions do show a different metabolic response to virus infection. Also for other viruses, results concerning glycolytic activity after infection were not consistent according to different publications [20,21].

**Table 4:** Specific uptake and release rates of extracellular metabolites for infected (variant from NIBSC) and mock-infected cells.

	Cell specific rate [fmol/cell/h]					
	**Glucose**		**Lactate**		**Glutamate**	

	***infected***	***mock-infected***	***infected***	***mock-infected***	***infected***	***mock-infected***

Exp 2	-66	-45	667	295	20	-2
Exp 3	-255	n.d.	810	n.d.	17	n.d.

The measured amino acid concentrations did not reveal an impact on uptake or release rates with exception of glutamate, proline, serine and histidine. The effect of glutamate release upon influenza virus infection is well known [37-39]. For the infection with the RKI variant in Exp 1, however, no glutamate was released within the chosen time span according to the estimated rates (see Table 1). Nevertheless, the last two sampling points indicated a concentration increase (see Figure 2). Thus, also comparing to Exp 2 and Exp 3, the sampling time was not sufficiently long to measure a significant glutamate excretion.

A further comparison with literature appeared difficult, because different virus strains and lower MOIs were investigated there. Moreover, cultivations were performed in bioreactors. But most importantly, the work presented here was focused on the differences between infected and mock-infected cells, whereas no mock-infections were performed in the relevant literature.

Virus and mock-infected cells showed comparable rates until about 8-12 h pi, which is also the time point of virus release. Since virus production starts several hours before first virus release the energetic demands for production of virions can obviously not be detected with the performed measurements. Further, it can be assumed that the energetic demands for virus replication remain fairly constant also after the release. Therefore, the observed differences between infected and mock-infected cells after 8-12 h pi are assumed to originate from an induction of anti-viral mechanisms or the onset of degenerative processes related to virus-induced apoptosis rather than energetic demands for virus replication.

### Intracellular metabolite concentrations

Intracellular concentration changes are observed in infected as well as mock-infected cells (see Figures 4, 5, 9 and 10). This non-constant behavior was most likely caused by the exchange of medium at time of infection and the preceding washing of cells. On the one hand, effects on intracellular metabolite concentrations of new medium have to be expected due to high concentration of substrates (glucose, glutamine etc.) as well as low concentrations of waste products (lactate and ammonia) of the new medium. On the other hand, VMM did not contain serum. This alternation compared to growth medium should also affect cellular metabolism. Since main interest was in the difference between infected and mock-infected cells, time courses of metabolite concentrations of mock-infected cells will not be discussed in detail.

Throughout most of the measured metabolites, concentration changes occurred in infected cells. The time point of metabolic switch appeared to be different for both virus variants under investigation. Within the RKI experiments the metabolites in Exp 1 showed a later switch than in Exp 2. The concentration of the metabolite F16bP, for example, started to increase for Exp 1 earliest after 15 h pi, whereas for Exp 2, this concentration was significantly higher for infected cells compared to mock-infected cells at 12 h pi. More evident was the time difference between Exp 1 and Exp 2 for the ATP concentration. While for Exp 2 first concentration differences between mock-infected and infected appeared about 8 h pi, the ATP concentration did not drop earlier than 18 h pi in Exp 1. One possible explanation of this phenomenon could be that the infection of cells did not proceed identically. This is also reflected in the later start of cell death in Exp 1. As described above both cell seeding and virus infection are ruled to a certain degree by stochastic events resulting in six-well plates and individual wells of six-well plates with not identical stages of cell growth and virus infection. Since main interest of our work was in general effects of infection on metabolism, we combined the data sets of Exp 1 and Exp 2 for spline interpolations. These splines were assumed to yield a representative interpolation as well as a good estimate for the time point of the metabolic switch.

For the intermediates of the upper glycolytic part increased concentrations were found for infected cells for both virus strains tested. One open question remains concerning the concentration drop of sugar phosphates for the NIBSC virus after about 14 h pi. One possible explanation could be that two waves of infection occurred in the MDCK cells due to the comparatively low MOI for the virus from NIBSC (3 versus 20 for RKI). However, the fact that many metabolite concentrations showed a low value for the sample at 14 h pi supports the hypothesis that a sampling or dilution error was the reason for this phenomenon. Independently of the specific reason, the obvious trend of higher concentrations of sugar phosphates for infected cells can hardly be questioned.

Thus, the higher flux through glycolysis coincided with higher concentrations of the respective intermediary sugar-phosphates. According to textbook knowledge, the enzyme phosphofructokinase (PFK) is one of the rate-limiting steps of glycolysis [40,41]. Under the tested conditions, however, the metabolites F6P and F16bP, which are converted into each other by PFK, correlated well under the tested conditions (correlation coefficient of 0.89). This indicates that the differences in fluxes were probably not due to changes in the activity of the PFK. The next metabolite from glycolysis measured was 3PG. Also a slightly higher concentration for this metabolite for the infected cells was measured, even if the differences between infected and mock-infected cells were clearly smaller than for the preceding metabolites. Most interestingly, the concentration of PEP was significantly lower for infected cells for both virus strains under investigation. One possible mechanism leading to this phenomenon is the activation of the enzyme pyruvate kinase (PK) by the metabolite F16bP, a well known mechanism of metabolic regulation [40,41]. The inverse concentration behavior of F16bP and PEP in dependence of the glycolytic flux, as shown in the work presented here, was previously described after pulse experiments for both yeast and tumor cells [42-45].

The concentration of pyruvate showed an increase for both mock-infected and infected cells. The rate was slightly higher for cells infected with the RKI but also for the NIBSC virus. This corresponds well with the concentrations behavior of extracellular lactate. Also this concentration increased more strongly for the infected cells. The ratio of intracellular pyruvate and extracellular lactate remained fairly constant (approximately 0.03) after an initial drop caused by low extracellular concentrations of lactate for both mock-infected and infected cells, indicating a concomitant increase in both concentrations. According to literature, the ratio of intracellular concentrations of pyruvate to lactate can be used for the estimation of the [NAD^+^]/[NADH+H^+^] ratio [46,47]. As a further premise, however, it is necessary for this estimation to assume that intra- and extracellular lactate concentrations are in equilibrium [47]. Based on both assumptions, the physiologically important ratio of [NAD^+^]/[NADH+H^+^] does not seem to be affected by an infection of MDCK cells with influenza virus.

Taking both extra- and intracellular data together raises the questions on why the glycolytic flux is raised during infection and which regulatory mechanisms lay behind these observations.

Flux determining enzymes for glycolysis were often determined to be HK, PFK and PK for both continuous and cancer cell lines [48,49]. PFK seems not to be the rate-limiting step in the case of MDCK cells as described above. The high correlation between the first three glycolytic intermediates does not reveal any regulation at the point of PFK. It rather appears that HK controls the glycolytic flux. Ongoing work of our group on enzyme activities in MDCK cells showed that HK has during typical batch-cultivations the lowest activity of glycolytic enzymes (data not shown). Interestingly, especially the mitochondrial bound isoform of HK is described to be strongly involved in anti-apoptotic mechanisms of cells [11-13]. Thus, one hypothesis based on our results is that after viral infection, an increased expression of this enzyme occurs as an anti-apoptotic mechanism. As a by-product, the rate of glycolysis and intermediate concentrations are increased because HK represents the rate-limiting enzyme for glycolysis.

Measured concentrations of intermediates of the TCA-cycle were after 12 h pi for most metabolites higher for infected cells than for mock-infected cells. Concentrations of citrate and isocitrate correlated well (correlation coefficient of 0.86), which is in accordance with previous results showing an equilibrium of the reaction catalyzed by the enzyme aconitase (not shown). Concentrations of *α*-ketoglutaric acid appeared to be slightly higher for infected cells between 5 to 16 h pi, but due to general variability of data for this metabolite, differences were not considered significant. Also for succinate, no clear differences between mock-infected and infected cells were found. The concentration behavior of fumarate and malate correlated well (correlation coefficient of 0.77) underlining the high activity of the enzyme fumarase, typically stated in literature [40,41]. After about 4 h pi, intracellular concentrations for infected cells were slightly higher for both metabolites. If these increases were significant, it would represent the first detectable metabolic change after infection. Overall, the results indicate that it is difficult to draw clear conclusions from metabolites from TCA-cycle. However, this appears to be a logical consequence of the many intersections and metabolic shortcuts between the different intermediates. Moreover, compartmentalization is not considered using the method for determination of intracellular metabolites described above.

For both virus strains (from RKI and NIBSC) a simultaneous decrease of concentrations of nucleoside triphosphates and a concomitant increase in nucleoside mono- and diphosphates was seen, clearly indicating an energetic burden for infected cells after about 12 h. This can be seen even more pronounced for the values of the energy charge, significantly dropping after about 12 h pi. Typical values under physiological conditions for the EC are in a range of 0.9-1 for mammalian cells in general [31,49,50], but also for MDCK cells [50]. Infected cells exhibited values of below 0.9 reaching down to values of even below 0.8. This observation indicates the serious impact of influenza virus infection on cellular metabolism. For an infection of Vero cells with reovirus, Burgener et al. could show a decrease of the EC to values of 0.5 within two days pi. The comparison to an influenza infection, however, may be regarded with care due to large differences in the replication mechanisms compared to reoviruses.

A detailed study on effects of viral infection on host cell metabolism was done by Munger et al. [22]. In a metabolomics approach quantifying 63 intracellular metabolites they investigated the metabolic changes in fibroblasts after an infection with cytomegalovirus. Comparably to our work, they found an increased rate of glycolysis coinciding with higher metabolite pools for glycolytic intermediates. The concentration of PEP, however, increased significantly in the experiments done by Munger et al, whereas in the work presented here a clear decrease was found.

Moreover, Munger et al. concluded from their results that higher concentrations of F16bP were a result of a higher activity of the enzyme PFK. They could prove their hypothesis by measuring enzyme activities. Concluding from our result that F6P and F16bP were highly correlated, no glycolytic regulation at the point of PFK is expected for an influenza infection in MDCK cells.

Munger et al. (2008) [23] extended the conclusions of their previous work [22] on infections with cytomegalovirus based on results of a kinetic flux profiling study. Most pathways of the central metabolism were found to be up-regulated, including glycolysis, TCA-cycle and pyrimidine nucleotide biosynthesis. They could show an increased glucose and glutamine uptake and lactate and glutamate release rates. Comparability to an infection with influenza virus is certainly limited due to large biological differences to cytomegaloviruses, nevertheless, main effects may be similar.

Besides the question, which pathway fluxes and metabolite concentrations are increased, the time point of the metabolic switch is of interest. An overview of the distribution of various switch-points for different metabolites is shown in Figure 6. Obviously, the average time for the metabolic switch is at about 11 h pi. Interestingly, depending on metabolite class, different average time points were found. This indicates that metabolism does not react simultaneously on infection, but rather in a serial adaptation. On average, glycolysis was affected at first, followed by TCA-cycle intermediates. Finally, nucleotide concentrations changed. It has to be mentioned, however, that due to the fitting procedure, outliers for switch-points normally occur, but are assumed not to severely influence the general conclusions.

The fitting of linear splines was omitted for the concentrations obtained for the experiments with the NIBSC variant. The reason was that the linear estimations were strongly influenced by extreme values of a few data points, and the fact that the switch to changed metabolism of infected cells occurred at approximately 8-10 h pi and only few data points were recorded until then. Thus, no meaningful estimation of the time point for the metabolic switch was achieved. Nevertheless, it can be easily recognized from the time course of metabolites like G6P, F16bP or ATP that the metabolic switch occurred earlier than for the RKI variant. This observation shows that timely differences in host cell metabolism coincide with the different replication dynamic of virus variants.

One hypothesis based on the presented results is that during early stages of infection no severe load on metabolism is to be expected by replication of virus, as calculated earlier in connection with the work presented here based on a metabolic flux model [34,35], but also as estimated based on other assumptions [4,7]. Effects observed mainly reflect cytopathic effects of infection. Influenza infection typically causes apoptosis of host cells [5,6]. Even though anti- or pro-viral molecular mechanisms might have an influence on host cell metabolism, it appears that the breakdown of mitochondrial membrane due to apoptosis has the main impact on cellular metabolism. After this event, cellular respiration can not proceed resulting in a sharp concentration drop of ATP. Concomitantly, glycolysis is increased to compensate for the energetic deficiencies. The activation of glycolysis could be mediated by the low EC, which is described to strongly activate catabolic enzymes [41]. Furthermore, we assume that enzymes from TCA-cycle are still active despite disruption of mitochondrial membranes, because no large impact on intracellular TCA-cycle intermediate concentrations was found and, moreover, glutamine uptake was not significantly altered. Since no cellular respiration can take place any more, reduced coenzymes accumulate. One possibility to oxidize the coenzymes is to excrete reduced metabolic intermediates. Thus, the high glutamate release might represent a strategy of the cells to keep the levels of oxidized coenzymes high. In agreement with this hypothesis is also the secretion of proline, which can serve as a sink for reduction equivalents. More difficult appears the metabolic explanation for the accumulation of the amino acids histidine and serine. The release of serine from infected cells could be connected to the measured higher intracellular concentration of 3PG and, moreover, to the glutamate release. Both substances are precursors for serine.

## Conclusions

In the work presented here, effects of an influenza virus infection on host cell metabolism were investigated. The methodological approach by metabolic profiling and modeling proved to be applicable to answer several open questions concerning the dynamics of extra- and intracellular metabolite concentrations after infections. Two different influenza H1N1 variants (PR/8/34; obtained from either RKI or NIBSC) induced qualitatively the same metabolic changes in host cells compared to mock-infected cells. In the early stage of infection, virus replication did obviously not induce major effects on metabolism. Approximately at the onset of cell death caused by apoptosis, the rate of glycolysis was significantly increased coinciding with clearly changed intracellular metabolite concentrations for glycolytic intermediates. TCA-cycle intermediates appeared to be less effected by infection, but also some concentrations were increased. Furthermore, intracellular nucleotide concentrations were markedly affected by influenza virus infection. The adenosine nucleotide ratio (Energy Charge) dropped to physiologically unusual values below 0.9, indicating the breakdown of metabolism after virus infection.

Based on these observations, we conclude that virus replication itself does not have a major impact on metabolism, but rather the onset of apoptosis causes this metabolic imbalance. One key event in the course of apoptosis is the disruption of mitochondrial membranes. As a result, cellular respiration stops forcing the cells into a compensation strategy to keep energetic status at a sufficiently high level. This results in an increased rate of glycolysis. In contrast, TCA-cycle seems to be operating close to normal conditions, as no differences in glutamine uptake and intracellular concentrations of intermediate metabolites could be measured. The concomitant accumulation of reduced coenzymes is counteracted by a release of glutamate.

Furthermore, we assume that enzymes from TCA-cycle are still active despite disruption of mitochondrial membranes, because no large impact on intracellular TCA-cycle intermediate concentrations was found and, moreover, glutamine uptake was not significantly altered. Since no cellular respiration can take place any more, reduced coenzymes accumulate. One possibility to oxidize the coenzymes is to excrete reduced metabolic intermediates. Thus, the high glutamate release might represent a strategy of the cells to keep the levels of oxidized coenzymes high.

The observed metabolic differences between the two virus variants (from RKI and NIBSC) reflected mainly the different replication behavior in MDCK cells, i.e. the RKI variant replicated more slowly resulting in a retarded cell death compared to the NIBSC variant. This characteristic behavior could also be seen in extra- and intracellular metabolite concentrations. Especially the start of metabolic changes due to virus-induced apoptosis was initiated later for the virus from RKI than for NIBSC. Despite the clear results, further investigations should be performed focusing on an integrative approach of various research fields especially signal transduction, transcriptional and translational regulation together with metabolism. Typical apoptosis markers should be used and the integrity of the mitochondrial membrane should be monitored to confirm the hypothesis raised from the metabolic observations.

Moreover, especially for the NIBSC variant, an infection with a higher MOI should be investigated. Therefore, also a higher sampling density in the early phase of infection might be useful to better characterize the time switch of metabolism after infection.

Nevertheless, it can be concluded from our work that influenza virus infection has its main impact on metabolism during late stages of the replication process, and that the early virus replication phase is not a critical burden for cellular metabolism.

## Methods

### Cell and virus culture

**Figure 11:**
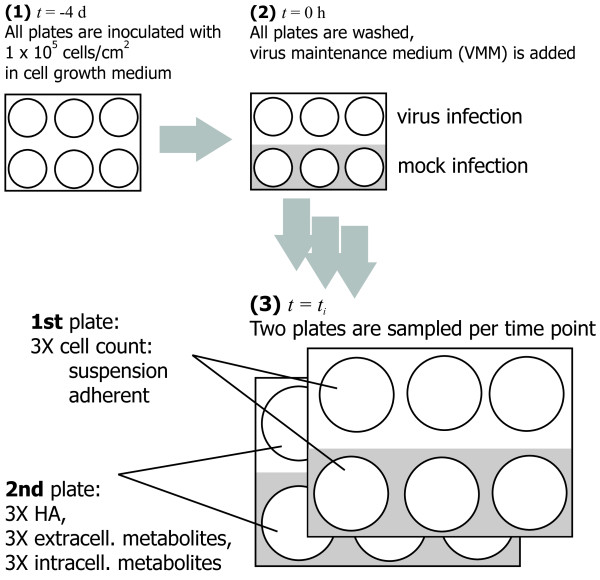
**Scheme of the experimental steps**. (1) MDCK cells are cultivated in 4 mL cell growth medium (GMEM with serum) for four days. (2) The confluent cell layer is washed and virus maintenance medium (VMM) is added, in case of infection with active virus particles. (3) Samples are taken from two six-well plates to monitor intracellular and extracellular concentrations.

Madin-Darby canine kidney (MDCK) cells (ECACC, # 84121903) were cultivated and infected as described previously [51] and depicted in Figure 11. Briefly, adherent MDCK cells were cultivated in GMEM supplemented with 10% FCS (Gibco, #10270-106) and 2 g/L peptone (International Diagnostics Group, #MC33) in six-well plates (Greiner Bio-One, # 657160) in an incubator at 37°C with 5% CO_2_. Cells were seeded four days prior to infection at a cell density of 7.5 × 10^4 ^cells/mL with a volume of 4 mL per well. Pre-cultures were cultivated in roller bottles (Greiner Bio-One, # 680XX).

MDCK cells were infected with MDCK cell-adapted human influenza A virus A/Puerto Rico/8/34 (H1N1) from the Robert-Koch-Institute (RKI, Berlin, Germany) or from the National Institute for Biological Standards and Control (NIBSC, Hertfordshire, U.K.). These virus variants are referred as RKI and NIBSC variants in the following text. For virus culture, serum-free VMM containing about 2.0 × 10^-6 ^U/cell porcine trypsin (Gibco #27250-018) was used. The total volume of VMM (with trypsin) needed for one complete experiment was divided in half. One half was used for infections with virus (= 1^st ^half) the other half for mock-infections (= 2^nd ^half). Active virus stock was added to adjust the required multiplicity of infection (MOI) to the 1^st ^half. MOIs were calculated based on volumes, the number of adherent cells at time of infection and infectious virus titers determined by TCID_50_. For determination of cell numbers at time of infection, six wells of one six-well plate were counted and averaged. Before infection, cell growth medium was removed and cells were washed three times with phosphate-buffered saline (PBS; 8 g/L NaCl, 0.2 g/L KCl, 0.2 g/L KH_2_PO_4_, 1.15 g/L Na_2_HPO_4_). To each of three wells of one six-well plate, 2 mL of VMM (1^st ^half; with trypsin and virus; *infection*) were added and to each of the remaining three wells 2 mL of VMM (2^nd ^half; with trypsin but without virus; *mock-infection*) were added. After addition, all six-well plates were placed in the incubator. To achieve a one-step infection of all cells, MOI was chosen between 3 (NIBSC) and 20 (RKI) depending on maximum titers of available virus seeds (2.1 × 10^7 ^virions/mL, NIBSC; 3.2 × 10^9 ^virions/mL, RKI).

### Sampling Procedure

At each sampling time point, two six-well plates (two for infection, two for mock-infection) were used for quantifications. The first plate was used for determination of suspension and adherent cell numbers. The supernatant of the second plate was used for measurement of extracellular metabolite concentrations and virus titers; adherent cells were used for determination of intracellular metabolite concentrations. Consequently, three independent biological samples were available for averaging of each variable measured.

### Determination of cell numbers

The number of suspension cells was determined directly after sampling using a ViCell XR Cell Viability Analyzer (Beckman Coulter). No discrimination was made between viable and dead cells because detached cells are typically assumed to be dead or in a late apoptotic stage [10]. For determination of the number of adherent cells, wells were washed three times with PBS followed by a 30 min trypsin (porcine, 2.5%, 0.5 g/L; Gibco #27250-018) treatment. A cell scraper was used to assure complete removal of cells. Cells were counted using the ViCell XR. No discrimination of viable and dead cells was made because adherent cells typically show high viabilities independent of the infection state.

### Determination of extracellular metabolite concentrations and virus titers

The collected supernatant of the second plate of one sampling time point was treated for 4 min at 80°C for inactivation of infectious virions, and subsequently stored at -70°C until measurement of extracellular metabolites and viral titers. Concentrations of extracellular metabolites (glucose, glutamine, glutamate, lactate and ammonia) were determined using a Bioprofile 100 Plus Analyzer (Nova Biomedical). Viral titers were determined according to a hemaglutination assay according to Kalbfuss et al. [52].

Amino acids other than glutamine and glutamate (see above) were determined directly after appropriate dilution by anion-exchange chromatography with integrated pulsed amperometric detection (BioLC system, Dionex) according to Genzel et al. (2004) [37]. Applying this procedure, 14 extracellular amino acids were quantified.

### Determination of intracellular metabolite concentrations

Sample preparation for analysis of intracellular metabolite concentrations was performed as reported previously [50]. All solutions used were ice-cold. Briefly, cell layers in wells of six-well plates were quickly washed once with 1 mL 0.9% NaCl solution followed by an immediate addition of 600 *μ*L of a MeOH/CHCl_3 _solution (1:1) to each well. Thus, only metabolites of adherent cells were quantified. After a short period of shaking, 500 *μ*L of this solution were removed and added to 2 mL sample tubes containing 500 *μ*L CHCl_3 _(100%). Afterwards, 900 *μ*L of MeOH/Tricine solution (9:10, Tricine 3.8 mM) were added to each well. After a short period of shaking, the complete liquid phase was transferred to the before mentioned 2 mL sample tube already containing MeOH/CHCl_3_. Tubes were vortexed for approximately 20 s and than centrifuged for 5 min at 16000 g at 0°C. In a next step, the water phase was aspirated from the sample tube and transferred to a new 2 mL sample tube. As described above, a second extraction of the cell layer was performed by adding 800 *μ*L of the MeOH/Tricine solution (9:10, Tricine 3.8 mM) to each well and a short period of shaking. This solution was collected in the following step and transferred to the remaining CHCl_3_-phase of the first sample tubes, vortexed, and centrifuged (16000 g, 5 min, 0°C). The water phase was collected again and pooled with the water phase from the first extraction. Finally, samples were dried using a stream of air and stored at -80°C until measurement. Samples were dissolved in 600 *μ*L of Milli-Q water.

Samples for intracellular metabolites were analyzed by LC-MS. Chromatographic separation was performed using an anion-exchange chromatography system (BioLC system, Dionex) as described previously [53]. In addition to conductivity- and UV-detection, a single-quadrupole mass spectrometer (MSQ Plus; Thermo Finnegan) was connected in series after the other two detectors. Calibration was done using external standards (5-155 *μ*L to a total volume of 1000 *μ*L) plus one additional standard concentration at the low concentration end (2.5 *μ*L to 1000 *μ*L) for every metabolite. Concentrations of metabolite standards in standard stock solution are summarized in Table 2. During one measurement sequence, standards were randomly injected to compensate for possible trends. This resulted in a maximum ratio of sample/standard of 4:1. Peak area was used for quantification and, depending on the metabolite, a first or second-order polynomial was used for fitting the calibration curve. Due to heteroscedasticity over the calibration range for some metabolites, a weighted regression (*1/concentration*) was applied. To assure good quantification at low concentrations, calibration curves were forced through the origin for some metabolites. Details on quantification method for all metabolites monitored are given in Table 2. In total, 29 intracellular metabolites were quantified. Based on the concentration of each metabolite in 600 *μ*L (volume after dissolving of dried samples), the well-specific amount of each metabolite was calculated. To compensate for differences in cell numbers at time of infection, intracellular concentrations were calculated based on a cellular volume of 2.33 × 10^-12 ^L assuming circular shape and a diameter of 16 *μ*m. Based on this, cell numbers were estimated according to a mathematical model as described in the next section.

### Interpolation of intracellular metabolite concentrations

After washing and addition of virus seed two phases were assumed to describe the progress of infection (1) adaption to the new medium and (2) effects of the viral infection (Figures 4 and 5). For linear data interpolation a simplified spline approach with a breakpoint between (1) and (2) was applied to reflect these phases. Thus, the interpolation is characterized by three base points (*t*_*i*_, *c*_*i*_). Two time points were fixed at *t*_0 _= 0 h (start) and *t*_2 _= 22.5 h (end). Thus, for the approximation four parameters had to be determined *t*_1_, *c*_0_, *c*_1 _and *c*_2_. Of special interest was the time point *t*_1_, which reflects the time point of a change in the trend of the intracellular concentrations. To increase data point density the replicate experiments were evaluated in a combined approach. Parameter estimation was performed using an iterative optimization algorithm (fminsearch; MATLAB, Version 7.4, The Mathworks, 2008).

### Mathematical model for the estimation of cell numbers and metabolic rates

Aim of the mathematical model was to calculate the metabolic rates of subpopulations of cells (see below) and to provide a basis for cell specific calculations based on noisy experimental data, i.e. for intracellular concentrations. Based on previous work [54,55] a segregated model was developed. Experimental data showed significant differences in extracellular concentrations of infected and mock-infected cells about 8-10 h pi (see results). The infection itself is a much faster process - high amounts of intracellular viral proteins are detected as soon as 4 h pi [10]. Therefore, adherent cells were classified into (1) *uninfected cells (X_*uinf*_)*, (2) *infected cells (X_*inf*_) *and (3) *infected and metabolically modified cells (X_*mm*_)*. During the time course of infection cells lose their contact to the growth surface. In the following, these suspension cells are called *detached cells (X_*det*_)*. After washing and addition of the virus medium (not containing virus in case of mock-infection) cells adapt to the new cultivation conditions. Cells infected with active virus particles transform into infected cells with a rate *r*_*inf *_. It has been observed from a series of experiments using flow-cytometry (not shown) that some cells cannot be infected - the amount of these cells are reflected in the parameter *X*_*ui*_. Infected cells start to produce virions. The experimental results as well as the above mentioned (Introduction) theoretical calculations indicate that virus replication only poses a minor metabolic burden. A change in specific metabolic rates is only observed in the later phase of infection. Therefore, a delay time (*τ*_*mm*_) and a rate of transition (*r*_*mm*_) from infected to infected and metabolically modified cells were introduced. As a result of virus replication, cells undergo apoptosis and detach from the surface. The rate of detachment is described by the rate *r*_*det*_. Again there is a delay, reflected by the delay time *τ*_det_. Cells in suspension are known to lyse (rate *r*_*lys *_has been taken from other experiments [54]). Following model equations were used to describe the time course of infection:(1)

The specific growth rate (*μ*) during infection was assumed to be zero because of (1) the culture is confluent after 4 days and no space is left for growth (MDCK cells grow in a monolayer) and (2) the virus medium does not contain growth factors. Earlier experiments have shown that the MDCK cell line used for the investigations presented here cannot be adapted to grow in VVM (not shown). In case of a mock-infection, only three states were taken into account (unadapted, adapted and detached: *X*_*uadp*_, *X*_*adp*_, *X*_*det*_). Like in case of the infection, the growth medium was withdrawn and new medium (virus medium without virus) was added.

The cells adapt to the new environment - thus unadapted (*X*_*uadp*_) and adapted cells (*X*_*adp*_) were taken into account. For this condition no growth was assumed because (1) mock-virus medium does not contain growth factors and (2) the cell monolayer is already confluent (like in the case of a viral infection). Some detachment is observed and reflected in detached cells *X*_*det*_.(2)

The metabolic activity was modeled assuming quasi steady state rates. Depending on the cell's state a certain constant specific metabolic rate is reached, the time for metabolic change in the transition from one state to the other was assumed to be very short compared to the experimental duration. Based on the theoretical estimation of metabolic burdens from viral production the model was simplified to one rate for uninfected and infected cells (*r*_*met,1*_). As detached cells are still intact and contain enzymes they were also assumed to be metabolically active. For simplicity they were assumed to exhibit the same metabolic rates like metabolically modified cells (*r*_*met,2*_). Glucose, lactate, glutamate, glutamine and ammonia were taken into account. Virus particles are only released from adherent metabolically modified cells (late infection phase).(3)

### Measurement Model for Parameter Estimation

Because only adherent cells and cells in suspension could be measured, the following measurement equations were used in the parameter estimation:(4)

As described above, virus particles were measured using the HA assay (HA), the measurement model for virus particles gets:(5)

### Parameter Estimation procedure

All calculation were performed using the modeling software gPROMS (Process Systems Engineering Ltd., gPROMS Advanced User Guide, Release 3.1.5, 2008, London, United Kingdom). All delay times, and specific rates (with exception of *r*_*lys*_) and the concentrations *X*_*ui *_were estimated from the experimental data. Because of the high correlations between the parameters *r*_*inf*_, *r*_*mm*_, *r*_*det *_and the time delays *τ*_*inf *_and *τ*_*det *_the transition rates have been estimated and then fixed. This estimation was based on a simultaneously fit to all experiments (Exp 1 - Exp 3). The delay times as well as all metabolic rates could then be estimated for the single experiments with fixed transition rates.

A remark has to be made for the estimation of experiment 2. Here, missing HA measurements in the first 18 h pi had a negative influence on the estimation of the specific VP production rate. Therefore the HA measurements from the third experiment were also used in the evaluation of Exp 2.

## Authors' contributions

JBR and SAW designed and performed the experiments. SAW set up the models and evaluated the data. JBR and SAW interpreted the results and prepared the manuscript. SF assisted in the evaluation of data, YG and UR supported the evaluation and corrected the manuscript. All authors read and approved the final version.
